# Management of Hyperlipidemia: A Case Report

**DOI:** 10.7759/cureus.117014

**Published:** 2026-09-27

**Authors:** Jennifer Orleow, Amber Reed, Sophie Purke

**Affiliations:** 1 Clinical Nutrition, Notre Dame of Maryland University, Maryland University of Integrative Health, Baltimore, USA

**Keywords:** case report, familial hypercholesterolemia, hyperlipidemia, lifestyle management, nutrition, red yeast rice

## Abstract

Hyperlipidemia is an important cardiovascular risk factor, particularly in individuals with a strong family history of hypercholesterolemia and premature cardiovascular disease. Red yeast rice (RYR) contains monacolin K, a naturally occurring compound structurally similar to lovastatin, and has demonstrated lipid-lowering effects. This case describes a 50-year-old man with hyperlipidemia and clinical features raising suspicion for an inherited lipid disorder who declined statin therapy. Over 12 months, the patient implemented dietary and lifestyle modifications and used RYR and other nutritional supplements. Total cholesterol decreased from 243 to 187 mg/dL, low-density lipoprotein cholesterol from 166 to 114 mg/dL, and triglycerides from 123 to 76 mg/dL, while high-density lipoprotein cholesterol increased from 55 to 58 mg/dL. Fasting glucose decreased from 100 to 92 mg/dL, and HbA1c was 5.4% when measured at follow-up. No adverse effects from the nutritional supplements were reported during follow-up. This case describes improvement in lipid parameters during a multimodal nutritional, lifestyle, and supplemental intervention and highlights the need for further research to evaluate these approaches in individuals with hyperlipidemia.

## Introduction

Hyperlipidemia, particularly elevated low-density lipoprotein cholesterol (LDL-C), is an established risk factor for atherosclerotic cardiovascular disease [1]. Patients with clinical features suggestive of familial hypercholesterolemia (FH), including a strong family history of hypercholesterolemia and premature cardiovascular disease, may be at particularly elevated risk. Some patients may be hesitant to begin lifelong statin therapy despite recommendations from their healthcare providers. Red yeast rice (RYR) is a lipid-lowering nutraceutical containing monacolin K, a compound structurally similar to lovastatin, and has demonstrated LDL-C-lowering effects [2,3]. However, RYR products may vary in active monacolin content, and research regarding their long-term efficacy and safety remains limited [2,3].

FH is commonly evaluated using clinical criteria such as the Simon Broome and Dutch Lipid Clinic Network (DLCN) systems [4]. In this case, the patient’s untreated LDL-C of 166 mg/dL and family history of premature cardiovascular disease in a first-degree relative yield a DLCN score of 2, which falls below the threshold for possible FH. The patient also did not meet the lipid thresholds required for classification under the Simon Broome criteria. However, his presentation included bilateral xanthelasma, a clinical finding reported among patients with FH [1], as well as a strong family history that included premature cardiovascular disease in his father, a patient-reported maternal diagnosis of FH, and hypercholesterolemia requiring statin therapy in his brother. Medical records and lipid values for his mother and brother were unavailable; therefore, these findings could not be further quantified within formal diagnostic criteria. Collectively, these findings raised clinical suspicion for an inherited lipid disorder despite the absence of diagnostic confirmation. Genetic testing was not performed; therefore, FH remained a clinical consideration rather than a confirmed diagnosis.

The integrative approach used in this case incorporated nutritional and lifestyle interventions in an individual with hyperlipidemia and clinical features raising suspicion for an inherited lipid disorder. The patient followed a lower-carbohydrate diet (<130 g/day) emphasizing fiber-dense fruits, vegetables, whole grains, healthy fats, and fish, while reducing refined carbohydrates, added sugars, and ultra-processed foods. Eggs and dairy were continued, and dietary fat and cholesterol were not specifically restricted. Evidence suggests that dietary cholesterol does not necessarily correspond directly with serum cholesterol concentrations and supports consideration of overall dietary quality in cardiovascular risk management [5-7]. RYR and other nutritional supplements were used alongside these dietary changes and increased physical activity. As multiple nutritional, lifestyle, and supplemental interventions were implemented concurrently, the contribution of any individual component to the observed changes cannot be determined.

This report describes the clinical course of a 50-year-old man with hyperlipidemia and clinical features raising suspicion for an inherited lipid disorder who declined statin therapy and implemented nutritional and lifestyle interventions. The case is reported in accordance with the CARE Guidelines [8].

## Case presentation

Over 12 months, a 50-year-old man demonstrated improvements in cardiometabolic parameters, including total cholesterol, LDL-C, high-density lipoprotein cholesterol (HDL-C), and triglycerides (Tables 1-2). At Visit 1, the patient presented to his primary care provider (PCP) for an annual examination and reported developing xanthelasma on the medial aspect of both upper eyelids. His only medication was vitamin D3 (2,000 IU daily). Laboratory evaluation revealed an elevated total cholesterol concentration of 243 mg/dL, LDL-C of 166 mg/dL, and fasting glucose of 100 mg/dL.

**Table 1:** Timeline of patient evaluation, interventions, and clinical outcomes Chronological overview of primary care and nutrition visits, implementation of dietary, lifestyle, and supplement interventions, and follow-up laboratory assessments over 12 months. PCP: primary care provider, PRO: patient-reported outcomes, FH: familial hypercholesterolemia, RYR: red yeast rice

Visit	Visit type	Details
Visit 1	PCP annual exam	Lipid panel showed elevated total cholesterol (243 mg/dL) and blood glucose of 100 mg/dL (high-normal). PCP advised a low-fat diet, increased fiber intake, and periodic cholesterol rechecks. PCP reviewed the potential benefits of statin therapy. Patient elected to delay statin therapy and focus on nutrition and lifestyle modifications.
Visit 2	Initial nutrition visit	Family history was significant for heart disease and hyperlipidemia (FH), treated with statins. Patient was interested in using nutrition (diet and nutritional supplements) and lifestyle modifications as an initial management approach. Recommended initiating dietary modifications and nutritional supplements (RYR, 1,200 mg; CoQ10, 200 mg; and omega-3 fish oil, 1,280 mg).
Visit 3	Follow-up nutrition visit	PRO: Patient reported feeling healthier. He joined a gym and lost 5 pounds. He changed his diet to mostly whole-food meals and reduced eating out to no more than twice per month. PCP follow-up was planned for mid-summer to recheck lipids.
Visit 4	PCP six-month follow-up lab evaluation	PCP also recommended increasing RYR to 2,400 mg.
Visit 5	PCP annual exam	Laboratory evaluation included lipid and metabolic panels. Total cholesterol decreased, and fasting blood glucose was within the normal range.
Visit 6	Final follow-up nutrition visit	Cholesterol continued to decrease. RYR was reduced to 1,200 mg, and the brand was switched to Designs for Health due to concerns about citrinin. Patient lost 10 pounds and planned to follow up as needed.

**Table 2:** Serial laboratory measurements obtained over 12 months Laboratory values show changes in lipid parameters, glycemic markers, liver enzymes, renal function markers, and other routine chemistry measurements during dietary, lifestyle, and supplement interventions. HDL-C: high-density lipoprotein cholesterol, LDL-C: low-density lipoprotein cholesterol, VLDL-C: very-low-density lipoprotein cholesterol, HbA1c: hemoglobin A1c, ALP: alkaline phosphatase, AST: aspartate aminotransferase, ALT: alanine aminotransferase, BUN: blood urea nitrogen, eGFR: estimated glomerular filtration rate

Laboratory marker	Visit 1	Visit 4	Visit 5	Reference range	Trend
Albumin	4.7	-	4.6	3.5-5.0 g/dL	Stable
ALP	77	-	75	44-147 IU/L	Stable
ALT	19	-	17	7-56 IU/L	Normal
AST	19	-	20	10-40 IU/L	Stable
Bilirubin, total	0.7	-	0.8	0.2-1.2 mg/dL	Stable
BUN	15	-	16	7-20 mg/dL	Stable
Calcium	9.7	-	9.7	8.6-10.2 mg/dL	Stable
Chloride	103	-	104	96-106 mmol/L	Stable
CO₂	23	-	26	20-29 mmol/L	Stable
Creatinine	1.13	-	0.93	0.76-1.27 mg/dL	Improved
eGFR	79	-	99	≥60 mL/min/1.73 m²	Improved
Glucose (fasting)	100	-	92	70-99 mg/dL	Improved
HbA1c	-	-	5.4	4.0-5.6%	Normal
Potassium	4.2	-	4.8	3.5-5.2 mmol/L	Stable
Protein, total	7.0	-	6.9	6.0-8.5 g/dL	Stable
Sodium	141	-	142	134-144 mmol/L	Stable
HDL-C	55	53	58	>40 mg/dL	Improved
LDL-C	166	133	114	<100 mg/dL	Improved
Total cholesterol	243	223	187	<200 mg/dL	Improved
VLDL-C	22	17	14	5-40 mg/dL	Improved
Triglycerides	123	93	76	<150 mg/dL	Improved

At Visit 2, the initial nutrition consultation, the patient reported that his father had experienced premature cardiovascular disease before age 55, his mother had reportedly been diagnosed with FH, and his brother had hypercholesterolemia treated with statin therapy. Nutritional recommendations included limiting carbohydrate intake to <130 g/day, targeting approximately 30 g of fiber daily through fruits, vegetables, and whole grains, reducing refined carbohydrates and added sugars, and emphasizing fish and healthy fat sources. Rather than restricting total fat, the nutritionist emphasized dietary fat quality. Dietary cholesterol was also not specifically restricted, consistent with evidence that dietary cholesterol intake does not directly correspond with serum cholesterol concentrations due to homeostatic regulation of cholesterol absorption and endogenous synthesis [7]. Increased physical activity was recommended. The nutritionist also recommended that the patient consider a trial of RYR and review this recommendation with his PCP. The patient subsequently began RYR at 1,200 mg/day and CoQ10 at 200 mg/day (NOW Supplements), as well as 1,280 mg/day of omega-3 fatty acids (Nordic Naturals).

At Visit 3, a nutrition follow-up visit, the patient reported adhering to the dietary recommendations, eliminating ultra-processed foods, reducing added sugar intake to less than 25 grams per day, and preparing meals at home. He was exercising regularly and had lost weight. He reported no side effects from the nutritional supplements. Overall, he reported increased energy levels and was eager to recheck his cholesterol levels.

At Visit 4, his PCP obtained a six-month lipid recheck. Total cholesterol had decreased to 223 mg/dL, LDL-C to 133 mg/dL, and triglycerides to 93 mg/dL; HDL-C was 53 mg/dL, and VLDL-C was 17 mg/dL. The PCP recommended increasing RYR from 1,200 mg/day to 2,400 mg/day and returning in another six months for repeat laboratory studies. At Visit 5, the patient returned to his PCP for his annual examination, which included laboratory studies. Total cholesterol had decreased to 187 mg/dL, LDL-C to 114 mg/dL, and triglycerides to 76 mg/dL; HDL-C was 58 mg/dL. Fasting glucose was 92 mg/dL, and HbA1c was 5.4%. The patient reported continued adherence to the dietary recommendations described above and exercising six days per week. He had also increased his consumption of fatty fish to two to three times per week, eliminated alcohol, and reduced stress with a renewed focus on work-life balance. He reported discontinuing the omega-3 supplement.

At Visit 6, the nutritionist reviewed the patient's dietary and lifestyle recommendations, reported adherence to these recommendations, and laboratory results. Given the improvement in lipid parameters and the patient's preference to minimize supplement use, the nutritionist recommended reducing RYR from 2,400 mg/day to 1,200 mg/day. The patient had expressed concern regarding potential citrinin exposure from RYR products; therefore, a Designs for Health RYR product was selected based on the manufacturer's quality-control testing for citrinin. Potential adverse effects associated with RYR use (e.g., muscle pain or weakness, gastrointestinal symptoms, headache, and dizziness) were monitored through patient-reported symptoms during follow-up, with no adverse effects reported. The nutritionist also recommended restarting omega-3 fatty acids, which the patient had independently discontinued for an undocumented reason, and continuing vitamin D3 and CoQ10.

## Discussion

Although the patient did not restrict dietary cholesterol, he experienced significant improvements in total cholesterol, LDL-C, HDL-C, triglycerides, and fasting glucose while following a lower-carbohydrate, high-fiber diet rich in fruits, vegetables, whole grains, healthy fats, and fish [5]. Emerging evidence suggests that dietary cholesterol, particularly from eggs, may not significantly influence blood cholesterol levels or cardiovascular outcomes [5,7].

Nutritional supplements, including RYR, have been studied for their potential role in lipid management. Evidence from randomized controlled trials and reviews supports the LDL-C-lowering effects of RYR [2,3]. In this case, LDL-C decreased from 166 mg/dL at baseline to 133 mg/dL at six months and 114 mg/dL at 12 months while RYR was used as part of the multimodal intervention. However, because dietary and lifestyle modifications and other nutritional supplements were implemented concurrently, the independent contribution of RYR to the observed LDL-C reduction cannot be determined.

Omega-3 fatty acids have been studied for their triglyceride-lowering and potential anti-inflammatory effects. In patients with FH, omega-3 supplementation has been shown to influence the postprandial metabolism of apolipoprotein(a) [6,9]. Other research suggests that omega-3 supplementation may lower triglycerides in patients with FH but has not demonstrated improvement in endothelial function. At the same time, adherence to a Mediterranean dietary pattern may provide broader cardiovascular benefits [10]. In the present case, the patient initially used an omega-3 supplement but independently discontinued it before the final laboratory assessment. During this period, he increased his consumption of fatty fish to two to three times weekly, providing a whole-food source of omega-3 fatty acids consistent with the dietary intervention. Therefore, the observed lipid changes cannot be attributed specifically to omega-3 supplementation.

Regular aerobic exercise may reduce triglycerides, total cholesterol, and LDL-C while increasing HDL-C [11]. Regular exercise may also improve endothelial function and reduce systemic inflammation [4]. In FH populations, Tada et al. [12] found that adherence to a healthy lifestyle, including regular physical activity, was associated with a lower risk of coronary artery disease (CAD). Similarly, Fahed et al. [13] reported an association between healthy lifestyle practices and lower CAD risk among individuals with FH. In the present case, increased physical activity was recommended as part of the lifestyle intervention, leaving exercise modalities to patient preference. The patient subsequently reported exercising six days per week, although the specific exercise modalities were not systematically documented.

In this case, the patient's lipid profile improved with dietary changes, nutritional supplements, and lifestyle modifications, with LDL-C and triglycerides decreasing by 31% and 38%, respectively. RYR and omega-3 fatty acids were used during portions of the intervention, while the patient also increased his consumption of fatty fish and made other dietary and lifestyle changes. The intervention also included regular physical activity, and the patient reported exercising six days per week. Over the intervention period, HDL-C increased by 5.5%, and the patient lost 10 pounds. Regular exercise has been associated with favorable changes in lipid parameters [11]; however, because multiple interventions occurred concurrently, the contribution of exercise or any other individual component to the observed changes cannot be determined. Together, these interventions addressed multiple aspects of lipid and cardiovascular risk management through dietary modification, nutritional supplementation, and physical activity.

A strength of this case report is the longitudinal documentation of the patient's clinical course and serial laboratory measures over 12 months, during which lipid parameters and fasting glucose improved without reported adverse effects from the nutritional supplements. The case also describes an individualized approach to managing hyperlipidemia in a patient with clinical features raising suspicion for an inherited lipid disorder. A limitation of this case report is its focus on a single patient, which limits the generalizability of the findings. Although the patient's presentation raised suspicion for FH, available lipid values did not meet formal diagnostic criteria, genetic testing was not performed, and complete family records were unavailable. Consequently, FH remained a clinical consideration rather than a confirmed diagnosis in this case.

Additionally, multiple dietary, lifestyle, and supplemental interventions were implemented concurrently, making it impossible to determine the independent contribution of any single intervention to the observed outcomes. Lp(a) was not measured, limiting assessment of this component of cardiovascular risk. Measurement of Lp(a) could further refine cardiovascular risk assessment and inform future management. Future research with larger populations and more comprehensive biomarker assessment will be necessary to further evaluate these approaches to managing hyperlipidemia.

## Conclusions

This case describes an integrative approach to hyperlipidemia management that combines nutritional recommendations, including diet and supplements, with lifestyle modifications. The patient's family history and clinical presentation raised suspicion for FH, although the diagnosis was not confirmed. This case suggests that nutritional and lifestyle interventions may be part of an individualized approach to hyperlipidemia management, while recognizing that evidence-based pharmacologic therapy remains indicated when clinically appropriate. In this patient, lipid parameters improved while he followed a lower-carbohydrate, high-fiber diet rich in fruits, vegetables, healthy fats, and fish; continued to consume eggs and dairy; used nutritional supplements; and increased physical activity. Over 12 months, total cholesterol, LDL-C, triglycerides, and fasting glucose decreased, while HDL-C increased; HbA1c was 5.4% when measured at follow-up. These findings occurred without restricting dietary cholesterol or total fat. However, because multiple dietary, supplemental, and lifestyle interventions were implemented concurrently, the observed changes cannot be attributed to any individual component. Larger studies are needed to further evaluate these approaches to hyperlipidemia management.
